# Plasma-Based Proteomics Profiling of Patients with Hyperthyroidism after Antithyroid Treatment

**DOI:** 10.3390/molecules25122831

**Published:** 2020-06-19

**Authors:** Afshan Masood, Hicham Benabdelkamel, Aishah A. Ekhzaimy, Assim A. Alfadda

**Affiliations:** 1 Proteomics Resource Unit, Obesity Research Center, College of Medicine, King Saud University, P.O. Box 2925 (98), Riyadh 11461, Saudi Arabia; afsmasood@ksu.edu.sa (A.M.); hbenabdelkamel@ksu.edu.sa (H.B.); 2 Department of Medicine, College of Medicine and king Saud Medical City, King Saud University, P.O. Box 2925 (98), Riyadh 11461, Saudi Arabia; aishahekhzaimy@hotmail.com

**Keywords:** hyperthyroidism, plasma proteomics, inflammation, acute phase proteins, apolipoproteins, hypercoagulability, carbimazole, ingenuity pathway analysis

## Abstract

Thyroid hormones critically modulate body homeostasis and haemostasis by regulating energy and metabolism. Previous studies have focused on individual pathways or proteins that are affected by increases in thyroid hormone levels, while an overall plasma proteomic signature of this increased level is lacking. Herein, an integrated untargeted proteomic approach with network analysis was used to identify changes in circulating proteins in the plasma proteome between hyperthyroid and euthyroid states. Plasma from 10 age-matched subjects at baseline (hyperthyroid) and post treatment with carbimazole (euthyroid) was compared by difference gel electrophoresis (DIGE) and matrix-assisted laser desorption/ionization time of flight (MALDI TOF) mass spectrometry (MS). A total of 20 proteins were identified with significant difference in abundance (analysis of variance (ANOVA) test, *p* ≤ 0.05; fold-change ≥ 1.5) between the two states (12 increased and 8 decreased in abundance in the hyperthyroid state). Twelve protein spots corresponding to ten unique proteins were significantly more abundant in the hyperthyroid state compared with the euthyroid state. These increased proteins were haptoglobin (HP), hemopexin (HPX), clusterin (CLU), apolipoprotein L1 (APOL1), alpha-1-B glycoprotein (A1BG), fibrinogen gamma chain (FGG), Ig alpha-1 chain C region (IGHA1), complement C6 (C6), leucine rich alpha 2 glycoprotein (LRG1), and carboxypeptidase N catalytic chain (CPN1). Eight protein spots corresponding to six unique proteins were significantly decreased in abundance in the hyperthyroid samples compared with euthyroid samples. These decreased proteins were apolipoprotein A1 (APOA1), inter-alpha-trypsin inhibitor heavy chain 4 (ITIH4), plasminogen (PLG), alpha-1 antitrypsin (SERPINA1), fibrinogen beta chain (FGB), and complement C1r subcomponent (C1R). The differentially abundant proteins were investigated by ingenuity pathway analysis (IPA). The network pathway identified related to infectious disease, inflammatory disease, organismal injury and abnormalities, and the connectivity map focused around two central nodes, namely the nuclear factor kappa-light-chain-enhancer of activated B cells (NF-κB) and p38 mitogen-activated protein kinase (MAPK) pathways. The plasma proteome of patients with hyperthyroidism revealed differences in the abundance of proteins involved in acute phase response signaling, and development of a hypercoagulable and hypofibrinolytic state. Our findings enhance our existing knowledge of the altered proteins and associated biochemical pathways in hyperthyroidism.

## 1. Introduction

Thyroid hormones (THs) regulate a vast spectrum of central and peripheral metabolic functions that influence the homeostatic, metabolic, and regulatory activities of the major target organs, as well as the haematopoeitic system [1,2,3]. It is well known that THs maintain an individual’s overall energy balance, appetite, obligatory thermogenesis, and body weight [4,5] through modulation of the carbohydrate, lipid, protein, and intermediary metabolisms. They are also known to control the metabolic rate and oxygen consumption by altering membrane permeability and the expression of ion pumps [6]. These metabolic processes are mainly regulated by the unbound circulating TH fraction (1%) via ligand-mediated binding to nuclear thyroid hormone receptors (for the genomic action of THs) or peripheral integrin receptors (for the non-genomic-action of THs). Alternatively, the fraction of THs (99%) bound to plasma proteins have been considered to serve as an extrathyroidal pool necessary for their transport to target tissues where they undergo deiodination into the active triiodothyronine (T3), rather than serving any physiological purpose.

Recent studies have shown that, besides binding directly to the plasma proteins, THs can bind to components of the circulating blood and plasma via peripheral membrane integrin receptors present on the red and white blood cells, platelets, and vascular endothelial circulatory system constituents [7]. These non-genomic actions of THs directly influence cellular metabolism, regulation, and function. Disorders of the thyroid gland, resulting in increased free TH levels, lead to a hypermetabolic state, termed as hyperthyroidism, which clinically manifests as palpitations, fatigue, tremors, anxiety, disturbed sleep, weight loss, and heat intolerance. The degree of thyroid dysfunction is clinically assessed through biochemical measurements of the circulating levels of free thyroxine (T4) and thyroid-stimulating hormone (TSH) in the serum. Although these biochemical measurements are well established, they only reflect the central action and signaling of THs, while reliable molecular markers that can serve as indicators for the peripheral tissue actions of THs are non-existent [8].

The underlying molecular mechanisms of THs in humans remain to be elucidated, as much of the knowledge of metabolic regulation modulated by THs has largely been derived from animal models or in vitro cell culture studies, owing to limited accessibility to human samples. Blood, which is regarded as circulating connective tissue, represents an easily accessible human tissue, and measurements of the proteins within the circulatory system are known to mirror an individual’s physiology. In order to rapidly identify significant protein markers for determining the state of thyroid disease, we can use proteomics to bridge the gap between the encoded genome and its translation into proteins. A previous proteomic study by Pietzner et al. studied changes in the plasma proteome in an experimental human thyrotoxicosis model to screen for novel peripheral biomarkers of thyroid function [9]. Engelmann et al. experimentally induced thyrotoxicosis in healthy volunteers to explore the effects of excess amounts of levothyroxine (L-thyroxine, a synthetic form of thryoxine) on the plasma proteome, with a main focus on coagulation markers [10].

We have previously described the plasma proteome of hypothyroid patients with and without disease using an untargeted proteomic two-dimensional difference in gel electrophoresis (2D-DIGE) approach [11]. In the present study, we aimed to analyze the plasma proteome of the hyperthyroid patients during the disease state and after six months of anti-thyroid therapy in which they reverted to a normal euthyroid state. Understanding these changes in plasma proteome that occur during hyperthyroidism will further elucidate the peripheral and cellular effects of TH within the closed plasma protein environment and the haemostatic system. To identify the proteins that are potentially involved in hyperthyroidism, we used a proteomics-based approach that involves immune depletion of high-abundance proteins, 2D-DIGE analysis, and subsequent matrix-assisted laser desorption/ionization time of flight (MALDI TOF) mass spectrometry (MS) identification to obtain a panel of differentially expressed plasma proteins.

## 2. Results

### 2.1. Biochemical Parameters of the Study Subjects

The laboratory characteristics of the study participants are summarized in Table 1. Statistically significant changes (*p* < 0.001) were observed in the biochemical profiles of FT4 (free thyroxine) and TSH, as expected, and in the serum high-density lipoprotein (HDL) levels after anti-thyroid treatment.

### 2.2. Mass Spectrometry Identification of Differentially Expressed Proteins on 2D-DIGE

**Figure 1:**
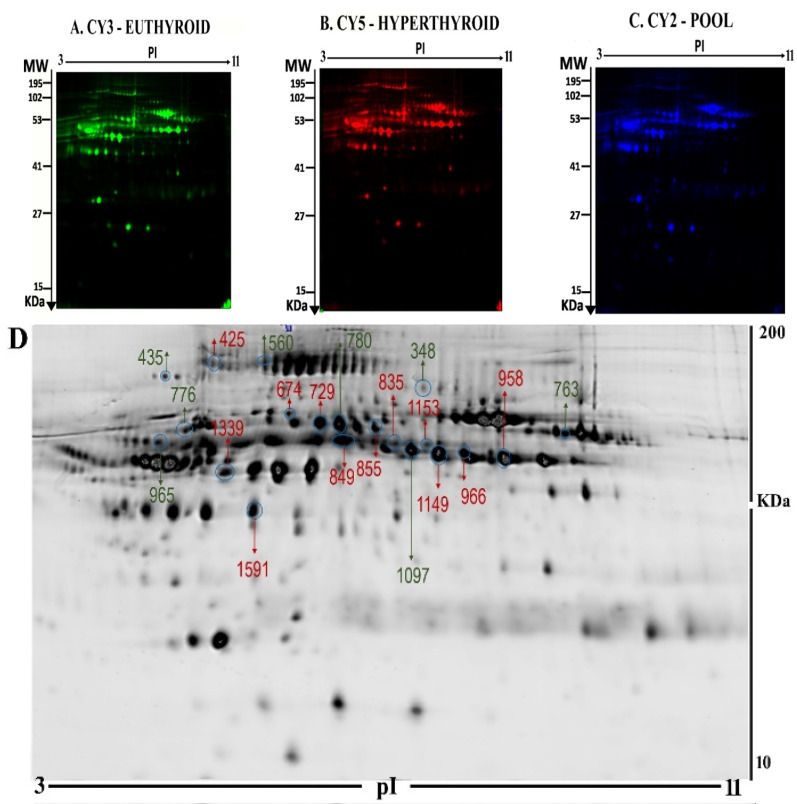
Representative fluorescent protein profiles of a two-dimensional difference in gel electrophoresis (2D-DIGE) containing euthyroid samples labelled with Cy3 (**A**), hyperthyroid labelled with Cy5 (**B**), and pooled internal control labelled with Cy2 (**C**). 2D-DIGE numbered spots indicate those proteins that were identified to be differentially abundant (defined as fold-change > 1.5, *p* < 0.05) between the hyperthyroid and euthyroid states and successfully identified with matrix-assisted laser desorption/ionization time of flight (MALDI TOF) mass spectrometry (MS) (**D**). MW, protein molecular weight; pI, isoelectric point.

2D-DIGE proteomics technology was used to investigate the differences in the pattern of protein level changes in the hyperthyroid samples (n=10) compared with the euthyroid samples (n = 10). The images were analyzed using the Progenesis Same Spots v.3.3 software (Nonlinear Dynamics Ltd., UK). After exclusion of false spots via rigorous manual validation, 945 spots were identified on the gels, of which 45 were significantly different (analysis of variance (ANOVA) p-value ≤ 0.05 and fold-change ≥ 1.5) between the hyperthyriod and euthyroid after treatment with antithyroid medication. Representative gel images are shown in Figure 1A–C, with differentially abundant spots indicated using arrows (Figure 1D). The spot patterns were reproducible across all ten gels, indicating that alignment and further analysis were possible. Cy2-labeling (the internal standard) was included to permit normalization across the complete set of gels and quantitative differential analysis of the protein levels (Table S1).

**Figure 2:**
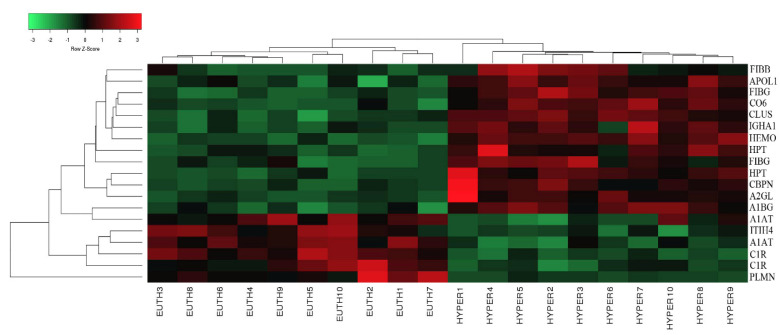
Heat map analysis representing the 20 significantly differentially abundant proteins between the hyperthyroid and euthyroid states after treatment with antithyroid medication. The horizontal line above the heat map represents the patients (euthyroid state (n = 10) and hyperthyroid state (n = 10)). Vertical lines represent the 20 significant proteins.

**Figure 3:**
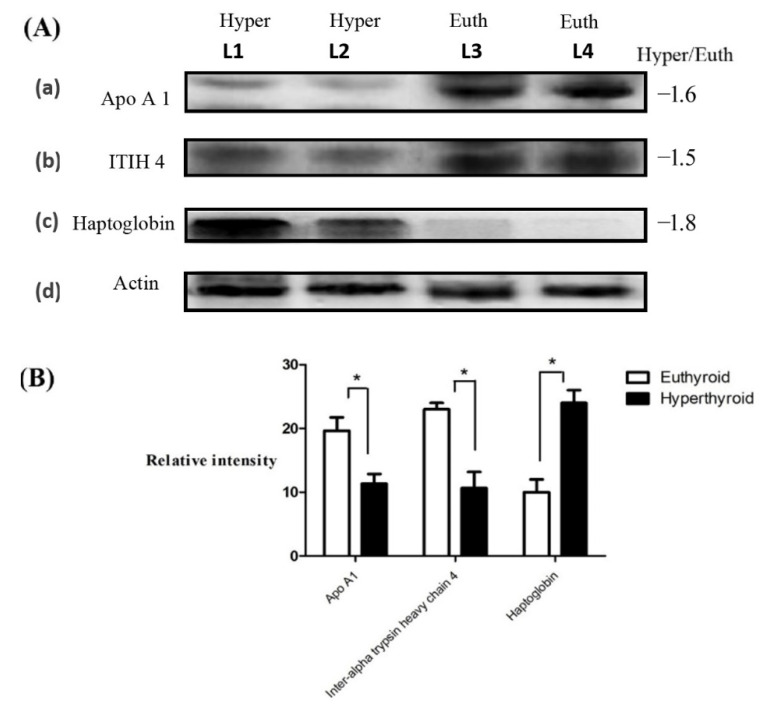
Confirmation of the proteomic data using immunoblot analysis of selected proteins (**A**). Western blot confirmation was performed for (**a**) ApoA1, (**b**) ITIH4, and (**c**) haptoglobin expression, as labeled in each panel. Lane: L1–L2 represent hyperthyroid and L3–L4 represent euthyroid states, respectively, duplicated. (**d**) Actin blots in the gel served as protein loading control. The gel pictures correspond to the same experiment and the blots were processed in parallel. The gel image was cropped and separated with black lines for each protein to ensure better understanding of the blot. Immunoblot results were similar to the results obtained by 2D-DIGE (**B**). Graphical representation of the relative intensity values of the normalized protein bands between the hyperthyroid and euthyroid states. The data are reported in histograms as mean ± SD.

From the 45 spots, 20 spots were successfully identified by peptide mass fingerprint (PMF), and were found to be unique protein sequences by MALDI-TOF mass spectrometry and matched to entries in the SWISS-PROT database by Mascot software with high confidence. The sequence coverage of the identified proteins by PMF ranged from 23% to 72%. In some cases, variants of the same protein were found at several locations on the gel (Table 1, Figure 1D). Twelve protein spots corresponding to ten unique proteins were significantly more abundant in the hyperthyroid samples compared with the euthyroid samples. These increased proteins were haptoglobin (HP), hemopexin (HPX), clusterin (CLU), apolipoprotein L1 (APOL1), alpha-1-B glycoprotein (A1BG), fibrinogen gamma chain (FGG), Ig alpha-1 chain C region (IGHA1), complement C6 (C6), leucine rich alpha 2 glycoprotein (LRG1), and carboxypeptidase N catalytic chain (CPN1). Eight protein spots corresponding to six unique proteins were significantly decreased in abundance in the hyperthyroid samples compared with euthyroid samples. These decreased proteins were apolipoprotein A1 (APOA1), inter-alpha-trypsin inhibitor heavy chain 4 (ITIH4), plasminogen (PLG), alpha-1 antitrypsin (SERPINA1), fibrinogen beta chain (FGB), and complement C1r subcomponent (C1R). Among the identified proteins, HP, FGB, and alpha-1-antitrypsin were found in more than one spot, which can be explained by their post-translational modifications, cleavage by enzymes, or the presence of different protein species. The heat map was generated using all 20 significant proteins identified by mass spectrometric analysis. The resulting heat map (Figure 2) showed differences in the protein abundances between the hyperthyroid and euthyroid state. The differential expression of three of these identified proteins (APOA1, ITIH4, and HP) hyperthyroid and euthyroid human plasma samples were validated using immunoblot analysis (Figure 3A–B). Immunoblot data were normalized using the housekeeping protein α-actin.

### 2.3. Interactions of Identified Proteins and Network Connectivity Mapping Using Ingenuity Pathway Analysis (IPA)

**Figure 4:**
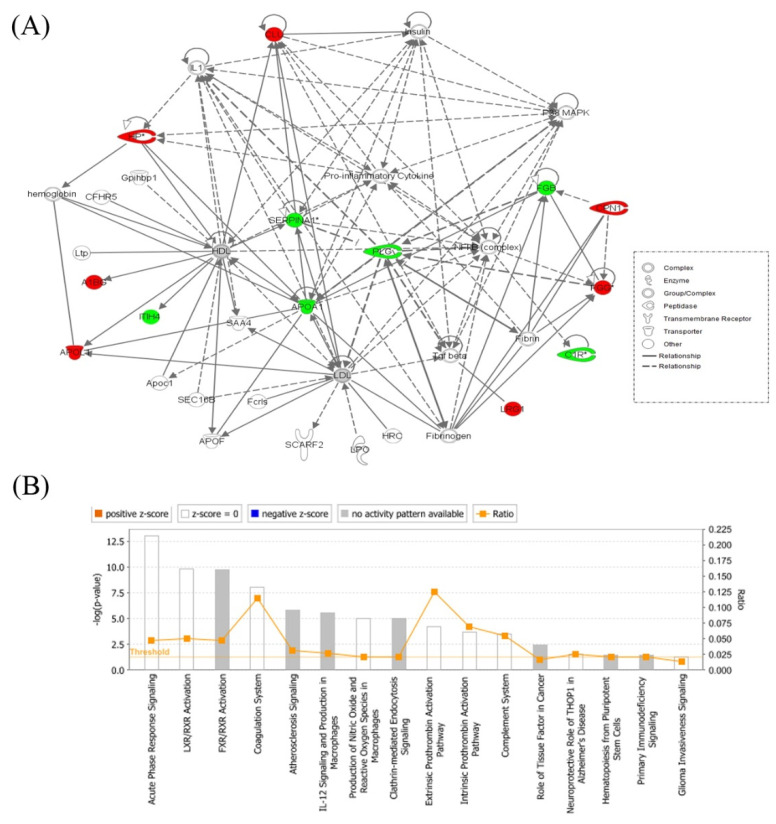
Schematic representation of the highest scoring network pathway depicting the involvement of the differentially regulated proteins between the hyperthyroid and euthyroid states. Ingenuity pathway analysis (IPA) found the functional interaction network of the proteins was related to “infectious disease, inflammatory disease, organismal injury, and abnormalities” and focused around nuclear factor kappa-light-chain-enhancer of activated B cells (NF-κB) and p38 mitogen-activated protein kinase (MAPK) signaling pathways as central nodes, which were deregulated in hyperthyroidism. Nodes in green and red correspond to downregulated and upregulated proteins, respectively. Colorless nodes were proposed by IPA and suggest potential targets functionally coordinated with the differentially abundant proteins. Solid lines indicate direct molecular interactions and dashed lines represent indirect interactions (**A**). The diagram showing the 16 top canonical pathways ranked by the p-values obtained by the IPA (**B**).

The proteins in the data set identified with significant differential abundances were probed using the IPA program. The program uses an algorithm that generates scores using the ingenuity knowledge database and attributes them to the corresponding best fit biological network pathways. This connectivity map is enriched with other interacting nodes that are entered by the software and the relationships denoted as direct or indirect, by continuous or discontinuous connecting lines, respectively. The network with the highest score between the hyperthyroid and euthyroid state related to “Infectious Disease, Inflammatory Disease Organismal Injury and Abnormalities” (Figure 4A) and involved 13 of the 20 identified proteins as focus molecules. Additionally, the first canonical pathway highlighted with significance belonged to acute phase response signaling (ratio = 0.047, *p* = 9.94 × 10^−14^) (Figure 4B). The other canonical pathways are summarized in Supplementary Figure S1.

### 2.4. Classification of Key Proteins ased on Function

**Figure 5:**
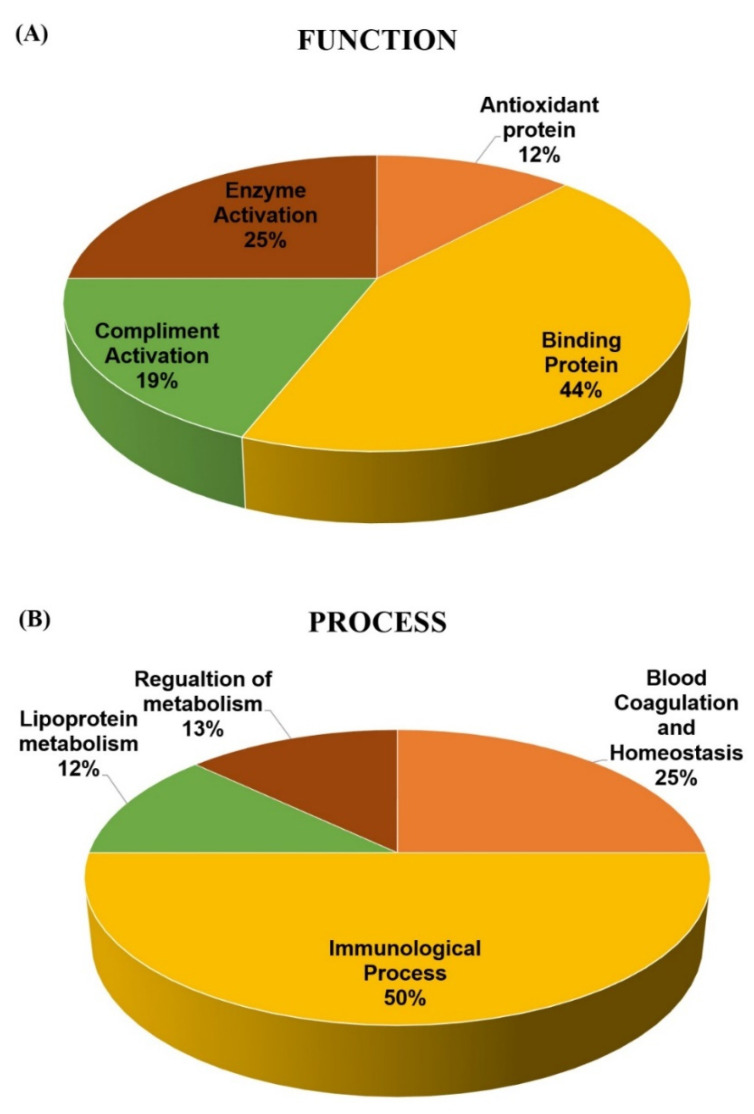
Comparative depiction of the differentially abundant proteins categorized into groups according to their function (**A**) and biological process (**B**). Protein functions and process were based on the Gene Ontology GO terms available on the UniProt Knowledge base. The representative pie chart shows graphically the percentage of identified proteins involved in each of the different functional categories.

To gain a better understanding of the functional role, localization, and biological process of the identified proteins, we used the UniProt gene ontology (GO) system for their annotation. The functional analysis of the differentially expressed proteins in our study revealed that they belong mainly to classes of enzymes, transport proteins, and immunoglobulins (Figure 5A). The majority of the identified plasma proteins, based on their location, were extracellular as expected (Figure 5B). The only exception was CLU, which is known to be present both intra- and extracellularly.

## 3. Discussion and Conclusions

Human metabolism crucially depends on adequate levels of TH for its optimum function. These occur via its central genomic as well as its peripheral, non-genomic actions at the level of the target tissues and cells, including cells of the circulatory and vascular systems. It is well known that THs are involved in modulation, secretion, and degradation of a multitude of plasma proteins. In hyperthyroidism, the latter process is assumed to be predominant owing to the presence of a hyper-metabolic catabolic state characterized by increased resting energy expenditure, lipolysis, gluconeogenesis, and weight loss, as well as reduced cholesterol levels [7].

In the present study, we characterized the differences between the plasma proteome of patients during the hyperthyroid state and after becoming euthyroid, following treatment with antithyroid medication, using an untargeted 2D-DIGE proteomics approach in combination with bioinformatic network analysis. We identified significant differences in the quantitative expression level of 20 protein spots, corresponding to 16 unique proteins in between them (Table 2).

The proteins identified with a significant increase in abundance between the hyperthyroid compared with euthyroid state were A1BG, HP, HPX, APOL1, C6, CLU, CPN1, FGG, IGHA1, and LRG1, while those with a significantly lower abundance were APOA1, SERPINA1, PLG, FGB, ITIH4, and C1R. These identified proteins are multifunctional in nature and are known to be involved in regulating multiple biochemical and metabolic pathways, individually or in conjunction with the other identified proteins. They are known to function as transport proteins, enzymes, regulators of metabolism, including blood haemeostasis, inflammation, and immunological processes. We broadly grouped these proteins based on their involvement in their major related biochemical pathways as those involved in (i) lipid and lipoprotein metabolism, (ii) the acute phase response (APR), and (iii) the vascular homeostasis and coagulation for ease.

### 3.1. Differential Regulation of Proteins Involved in Lipid Metabolism

Hyperthyroidism is associated with a decrease in the levels of total cholesterol, triglycerides, HDL, LDL, and apolipoproteins [12]. We found that six of the differentially abundant proteins identified in our study (APOL1, CLU, and HPX; as well as APOA1, PLG, and SERPINA1) are involved in the regulation of lipid and lipoprotein metabolism. APOA1 is known to participate in regulation of lipid and fatty acid metabolism independently and along with PLG, CLU, and APOL1 in reverse cholesterol transport; uptake; or with PLG, CLU, and SERPINA1 in lipid synthesis and release.

A decrease in the abundances of spots related to APOA1, the primary apolipoprotein of HDL, was noted in the hyperthyroid state, in line with the findings of Muls et al., who also demonstrated that the same [13] APOA1 is known to facilitate the HDL-mediated diffusion of TH through the cellular and intranuclear membrane for its genomic actions [14]. It is tempting to speculate that decreased APOA1 levels along with a concurrent decrease in HDL levels, as commonly seen in patients with hyperthyroidism, represent a regulatory mechanism aimed at preventing an excess in flow of both intracellular and intranuclear TH concentrations [14]. Moreover, APOA1, in association with PLG, acts as a key determinant of the cholesterol efflux capacity, a major process involved in reverse cholesterol transport (RCT) [15]. Decreased levels of both these proteins indicate that there is a decrease in RCT pathway in the hyperthroid compared with the euthyroid state, which predisposes the former to an increased risk of atherosclerosis and cardiovascular disease. The difference in the abundance of APOA1 between hyperthyroid and euthyroid states was independently confirmed using immunoblot analysis.

In addition to APOA1, protein spots relating to APOL1, a minor HDL3-associated apolipoprotein involved in lipid transport and metabolism, apoptosis, autophagic cell death, and cell lysis by membrane pore formation [16], were found to have significantly increased abundance in the hyperthyroid compared with the euthyroid state. Increased levels of APOL1 are known to be positively associated with hyperglycemia and plasma triglycerides in patients with coronary artery disease having low HDL, and are a potential factor for premature cardiovascular disease [17]. The increased levels of circulating plasma APOL1 have not been reported in relation to increased TH levels to date and need to be investigated in future studies.

We also observed an increase in the abundance of spot intensities relating to CLU (Apo J), another apolipoprotein, in the hyperthyroid state compared with the euthyroid. CLU is a secreted, multifunctional chaperone glycoprotein associated with HDL that, along with APOA1, helps in the RCT pathway. Lower CLU levels are known to be associated with insulin resistance, obesity, and dyslipoproteinemia, while higher levels are associated with coronary artery disease [18,19]. The concerted action of these proteins in regulation of HDL emphasizes the importance of alteration in HDL metabolism in the hyperthyroid state.

### 3.2. Differential Regulation of Proteins Involved in the Acute Phase Immune Response

In the present study, we also found a significant differential abundance in twelve of the identified proteins that function as acute phase proteins (APPs) and are involved in the regulation of acute phase response. Seven of them (HP, HPX, CLU, APOL1, FGG, A1BG, and LRG1) showed a significant increase in abundance, while another six showed a significant decrease in abundance (APOA1, PLG, SERPINA1, ITIH4, FGB, and C1R) in patients with hyperthyroidism before and after treatment.

Hyperthyroidism is characterized by increased levels of THs, of which T3 is known to increase the hepatic APPs [20]. APPs comprise a large, heterogeneous group of proteins and polypeptides whose concentrations increase during inflammatory states, largely in response to inflammation-associated cytokines. This relationship between these proteins and the pro-inflammatory cytokine was highlighted in the network pathway, which is deregulated in the hyperthyroid state. Classically, APPs are classified either as positive or negative APP based on their increased or decreased concentrations during inflammation. Recent studies have shown that the concentrations of specific APPs do not necessarily follow the same rule, and their varying concentrations are characteristic, reflecting the different disease conditions [11]. Increased TH levels have been shown to increase the concentration of APPs, including HP, A1BG, and fibrinogen and its end products, indicating an inflammatory state [21].

We observed an increase in spot intensities related to HP in the plasma of patients between hyperthyroid compared with the euthyroid state. HP is a positive APP that is known to scavenge free hemoglobin, prevent development of oxidative stress, and selectively bind APOA1. An increase in HP abundance, in the hyperthyroid state, may serve as an anitoxidant and to protect the diminishing APOA1 from further oxidative damage and loss of function [22]. In addition to these, HP functions as a regulator for vascular homeostasis and angiogenesis, and in regulating inflammation as previously described [11].

We also noted a decrease in abundance for the protein spot intensities relating to ITIH4 in the hyperthyroid state compared with the euthyroid. ITIH4 belongs to a group of IL-6-regulated serine protease inhibitors and is a known positive APP. It has been suggested to play a role in stabilization of extracellular matrix by binding to hyaluronic acid, may be involved in modulating inflammation and inhibit apoptosis. Previous studies have identified ITIH4 as a potential diagnostic and prognostic marker for a number of diseases that include acute ischemic stroke, ovarian cancer, interstitial cystitis, liver fibrosis [23] and its increased levels were seen in cases of thyroid cancer [24]. The decrease in the abundance of ITIH4 in our study was confirmed by immunoblotting.

Another serine protease inhibitor and a positive APP, SERPINA1, was identified with a decreased abundance in the plasma of hyperthyroid patients before and after treatment. SERPINA1 is known to possess the ability to not only inhibit serine proteases, such as collagenase, elastase, and proteinase-3, but also acts as an antioxidant and exerts anti inflammatory tissue-protective effects independent of protease inhibition. Decreased levels of SERPINA1 in hyperthyroid state may indicate a decrease in the inhibitory activity of the enzyme on the proteases and an increased inflammatory state [25]. Alternatively, elevated levels of SERPINA1 have been observed in inflammatory diseases and different types of malignancies, including thyroid cancer [26,27].

In addition to these proteins, circulating extracellular CLU, identified in our study with increased abundance, also acts as an anti-inflammatory agent. Its overexpression is known to attenuate the expression of proinflammatory chemokines, cell adhesion molecules, and matrix-degrading endopeptidases stimulated by the NF-κB signaling pathway [28], and its levels are elevated with oxidative stress and metabolic disease associated with systemic inflammation [29,30] similar to that seen in the hyperthyroid state.

Aside from the known APPs, we also identified C1R, a complement protein known to regulate the inflammatory process. C1R is a modular serine protease, a subunit of the complement initiating complex C1 and regulator of C1 activity and the complement cascade. The complement system not only acts as a rapid and efficient immune surveillance system, but is also involved in coagulation and the inflammatory process [31,32]. A decreased level of C1R suggests that increased TH levels predispose the patients with hyperthyroidism to a chronic inflammatory state [33].

### 3.3. Differential Regulation of Proteins Involved in Vascular Homeostasis and Coagulation

In the present study, we also found a significant differential abundance of proteins that influence vascular homeostasis, angiogenesis (SERPINA1, HP, and HPX), and the coagulation pathways (APOA1, PLG, SERPINA1, FGG, FGB, and IGHA1). It is known that increased circulating levels of TH, as seen in hyperthyroidism, impact proteins involved in the coagulation process, increasing the tendency for thrombosis accompanied by a parallel decrease in fibrinolysis [34]. Earlier studies have demonstrated that subclinical and iatrogenic hyperthyroidism are clinically associated with myocardial infarction, recurrent pulmonary embolism, and atrial fibrillation [35].

Recent studies indicate that endothelial cell dysfunction is an important pathogenic feature of hyperthyroidism that causes it to be associated with increased arterial stiffness (arteriosclerosis) [36]. The decrease in abundance of SERPINA1 (alpha 1 antitrypsin) identified in our study may also contribute to this pathology. The decrease levels of the enzyme lead to its decreased capacity to regulate neutrophil elastases, causing increased elastin degradation and increased collagen deposition in the endothelial cells, inducing arterial stiffness. This in turn creates a higher risk for developing arteriosclerosis [37], development of a more prothrombotic, procoagulant state leading to an increased risk of cardiovascular complications.

Another protein participating in the coagulation cascade and identified with decreased abundance in our study is PLG, an inactive precursor of plasmin found normally stored in platelets. Physiological concentrations of TH are known to act on platelets through their non-genomic mechanisms by binding to the peripheral receptors, resulting in degranulation, aggregation, and clot formation [38]. Activated plasmin is involved in clot lysis, while decreases in PLG levels are associated with thrombotic complications. The decrease in abundance of PLG detected in our proteomic study indicates the presence of a hypofibrinolytic state with an increased fibrin deposition and increased coagulation. Substantial clinical evidence indicates that TH modulates the complex process of blood coagulation and increases the risk of pathologic intravascular coagulation at different sites, including the brain (cerebral venous thrombosis) [39].

In addition to PLG, we also detected a significant differential abundance in two chains of fibrinogen glycoprotein, FGB and FGG, that is involved in coagulation cascade and tissue repair. Previous studies have shown a strong association between subclinical as well as overt hyperthyroidism with increased levels of fibrinogen, which shifts the haemostatic balance towards a hypercoagulable and hypofibrinolytic state. We found a decrease in the abundance of protein spot intensities relating to FGB, while an increase in abundance was noted for FGG. Marongiu et al. reported significantly increased plasma levels of fibrinogen, and specifically the Bβ chains in hyperthyroid patients that correlated with an increased risk of thromboembolism [40]. Lancellotti et al. on the other hand reported that the FGG chain reduces thrombin-induced platelet aggregation and is antithrombotic [41]. The differential regulation of these chains, decreased FGB, and increased FGG in our group of patients might point to compensatory regulation of the fibrinogen chains to reduce thrombotic events in our patients. Additional mechanistic studies need to be carried out to explore the molecular mechanisms and implications detail.

### 3.4. Network Pathway Analysis of the Significant Differentially Abundant Proteins

The bioinformatics and network pathway analysis was carried out using IPA to demonstrate the biological significance of the proteins and relate them to known pathways. The proteins identified in our study converged on two central nodes with highest connectivity; the nuclear factor kappa-light-chain-enhancer of activated B cells (NF-κB) and p38 mitogen-activated protein kinase (MAPK) signaling pathways. Both signaling pathways are known to be crucial for normal thyroid growth and function and for normal immune and inflammatory responses and are adversely affected in the case of thyroid autoimmune disease, thyroid cancer, and inflammation. NF-κB is a ubiquitous transcription factor involved in inflammatory and immune responses [42,43], as well as in regulating the expression of genes related to cell survival, proliferation, and differentiation Previous studies have identified the involvement of NF-κB in thyroid-specific gene regulation, thyroid autoimmunity, thyroid cancer, and thyroid orbitopathy, and vice versa. Increased NF-κB activity has been previously demonstrated in patients with hyperthyroidism and in livers from rats treated with exogenous T3 [20,43]. p38 MAPKs, on the other hand, are members of the MAPK family that are activated by a variety of environmental stresses and inflammatory cytokines. Involvement of the p38 MAPK signaling pathway suggests an increase in the non-genomic, rapidly acting thyroid signaling pathway that regulates cellular physiology, induces angiogenesis through hemodynamic effects, and promotes cell growth [44].

In summary, our proteomics analysis of the plasma of hyperthyroidism patients revealed changes in the abundance of several proteins involved in maintaining both homeostatic and hemostatic processes. Increased levels of circulating TH alter the transport and composition of lipids, lipoproteins, acute phase response proteins, and proteins involved in coagulation. Changes in the levels of these proteins can have serious clinical implications owing to endothelial dysfunction, the development of cardiovascular disease, and the increased risk of venous thrombosis or pulmonary embolism. Therefore, biochemical measurement of the proteins identified in our study could serve as clinical markers for the early detection of side effects related to the development of serious clinical complications in patients with hyperthyroidism. Future mechanistic studies are required to fully understand the clinical implications associated with changes in the levels of these differentially abundant proteins.

## 4. Materials and Methods

### 4.1. Ethical Considerations and Informed Consent

All procedures performed in the study involving human participants were in accordance with the ethical standards of the declaration of Helsinki and the universal International Conference on Harmonization-Good clinical practice (ICH-GCP) guidelines. The study protocol was approved by the Institutional Review Board, College of Medicine, King Saud University Hospital (No E-10-172). Written, informed consent was obtained from all participants.

### 4.2. Study Design and Subjects

We studied 10 patients (9 female and 1 male) with a mean age of 39.6 ± 10.6 years who were referred to our endocrine outpatient clinic at King Khaled University Hospital (KKUH) with newly diagnosed with overt hyperthyroidism. Blood samples were obtained from each patient after fasting for 10 h and before (pre-treatment sample) and after starting anti-thyroid medication (post treatment sample) The sample size was determined by carrying out a power analysis using the Progenesis SameSpots non-linear dynamics statistical software for determination of the minimum number of required biological replicates. Hyperthyroidism was defined as a FT4 (free thyroxine) levels higher than 22 pmol/L and TSH level lower than 0.25 mIU/L. Samples were obtained from each patient at two time points: pre-treatment samples (hyperthyroid state) were collected before starting anti-thyroid therapy and post-treatment samples (euthyroid state) were obtained from patients with normalized FT4 levels after treatment with the appropriate dose of antithyroid medication (carbimazole). None of the patients recruited in the study had any history of hypertension, type 2 diabetes mellitus, or other inflammatory or autoimmune disorders. Blood samples were collected after a standard 10 h fast using venipuncture into EDTA-coated tubes (Vacutainer, BD Biosciences, San Jose, CA, USA). The plasma was separated out after clotting by centrifugation (15 min, 3000 × g). The plasma was then divided into several aliquots and stored at −80 °C for further analysis.

### 4.3. Biochemical Analysis

All the parameters for biochemical and hormone analyses were carried out and determined using a Dimension Xpand Plus integrated clinical chemistry autoanalyzer (Siemens Healthcare Diagnostics, Deerfield, IL, USA) [45]. The serum levels of low-density lipoprotein (LDL) cholesterol were calculated using the Friedewald equation [46].

### 4.4. Sample Processing and Protein Extraction

Thawed plasma samples were centrifuged (5 min, 12,000 × g) and depletion of high-abundance plasma proteins (albumin, IgG) was carried out using an Albumin & IgG Depletion SpinTrap (GE Healthcare, Chicago, IL, USA) according to the manufacturer’s instructions. Protein extraction was carried out using Trichloracetic acid (TCA)/acetone precipitation, as described by Chen et al. [11]. The protein concentration of each sample was then determined in triplicate using the 2D-Quant Kit (GE Healthcare, Chicago, IL, USA).

### 4.5. CyDye labeling, 2D-DIGE and Imaging

Proteins were labeled according to the manufacturer (GE Healthcare, Chicago, IL, USA). Briefly, 50 µg of each hyperthyroid and euthyroid protein extract samples were minimally labeled with 400 pmol of the N-hydroxysuccinimide esters of Cy3 or Cy5 fluorescent cyanine dyes on ice for 30 min in the dark. A mixture of an equal amount of all samples was then pooled, labeled with Cy2, and used as an internal standard; this standard was normalized and matched across gels, dramatically decreasing gel-to-gel variation. A dye-switching strategy was applied during labeling to avoid dye-specific bias (Table S2, Figure S2). First-dimension analytical gel electrophoresis was performed, followed by second-dimension sodium dodecyl sulfate polyacrylamide gel electrophoresis (SDS-PAGE) on 12.5% fixed concentration gels, as previously described [11,46]. The gels were scanned with a Typhoon 9400 scanner (GE Healthcare, Chicago, IL, USA) using the appropriate wavelengths and filters for the Cy2, Cy3, and Cy5 dyes.

Differential in-gel electrophoresis (DIGE) images were analyzed using the Progenesis Same Spots v3.3 software (Nonlinear Dynamics Ltd., UK). The gel images were first aligned together and prominent spots were used to manually assign vectors to the digitized images within each gel. The automatic vector tool was next used to add additional vectors, which were manually revised and edited for correction if necessary. These vectors were used to warp and align gel images with a reference image of one internal standard across and within each gel. Gel groups were defined according to the experimental design, and the normalized volume of the spots was used to identify statistically significant differences. The software calculated the normalized volume of each spot on each gel from the Cy3 (or Cy5) to Cy2 spot volume ratio. The software performs log transformation of the spot volumes to generate normally distributed data. Log normalized volume was used to quantify differential expression. Independent direct comparisons were made between hypothyroid and euthyroid states, fold differences, and p-values were calculated using one-way ANOVA. All spots were pre-filtered and manually checked before applying the statistical criteria (ANOVA test, *p* ≤ 0.05 and fold difference ≥ 1.5). The normalized volume of spots, instead of spot intensities, was used in statistical processing. Only those spots that fulfilled the above-mentioned statistical criteria were submitted for MS analysis.

### 4.6. Colloidal Coomassie Blue Staining of the Preparative Gel

For the preparative gel, total protein (1 mg) was obtained from a pool of equal protein amounts of the 20 plasma samples. This sample was denatured in lysis buffer and then mixed in a rehydration buffer. Then, the proteins samples were separated by first and second dimensions with the same conditions in the DIGE section. Then, the gels were fixed in 40% (*v*/*v*) ethanol containing 10% (*v*/*v*) acetic acid (overnight) and then washed (3 ×, 30 min each, ddH2O). The gels were incubated (1 h, 34% (*v*/*v*) CH3OH containing 17% (*w*/*v*) ammonium sulphate and 3%(*v*/*v*) phosphoric acid) prior to the addition of 0.5 g/L Coomassie G-250. After 5 days, the stained gels were briefly rinsed with Milli-Q water and stored until the spots could be picked and identified by MS [47,48].

### 4.7. Protein Identification by MALDO-TOF MS

Coomassie-stained gel spots were excised manually, washed, and digested according to previously described methods [49]. The mixture of tryptic peptides (0.5 μL) derived from each protein was spotted onto a MALDI target (384 anchorchip MTP 800 μm Anchorchip; Bruker Daltonik, Germany) together with 0.5 μL of matrix (10 mg α-cyano-4-hydroxycinnamic acid (CHCA) in 1 mL of 30% CH3CN and 0.1% aqueous CF3COOH) and left to dry (room temperature, RT) before MS analysis. Spectra were acquired on a MALDI-TOF MS (UltraFlexTrem, Bruker Daltonics, Germany) in the positive mode (target voltage 25 kV, pulsed ion extraction voltage 20 kV). The reflector voltage was set to 21 kV and the detector voltage to 17 kV. Peptide mass fingerprints (PMF) were calibrated against a standard mixture by assigning appropriate mono-isotopic masses to the peaks; that is, bradykinin (1–7), m/z 757.399; angiotensin I, m/z 1296.685; angiotensin II, m/z 1046.54; rennin-substrate, m/z 1758.93; ACTH clip (1–17), m/z 2093.086; and somatostatin, m/z 3147.471 (peptide calibration standard II, Bruker Daltonics, Germany). MS spectra were recorded automatically across the mass range m/z 700–3000 and spectra were typically the sum of 400 laser shots. The PMFs were processed using Flex AnalysisTM software (version 2.4, Bruker Daltonics, Germany) and the sophisticated numerical annotation procedure (SNAP) algorithms were used for peak detection (S/N, 3; maximum number of peaks, 100; quality factor threshold, 30). MS data were interpreted using BioTools v3.2 (Bruker Daltonics, Germany), together with the Mascot search algorithm (version 2.0.04 updated 09/05/2018; Matrix Science Ltd., UK). Mascot parameters were as follows: fixed cysteine modification with propionamide, variable modification due to methionine oxidation, one missed cleavage site (i.e., in the case of incomplete trypsin hydrolysis), and amass tolerance of 100 ppm. Identified proteins were accepted as correct if they showed a Mascot score greater than 56 and *p* < 0.05, sequence coverage of at least 20%, and a minimum of four matched peptides. Not all spots of interest could be identified because some proteins were of low abundance and did not yield sufficiently intense mass fingerprints, whereas others were mixtures of multiple proteins [48].

### 4.8. Bionformatic Analysis

Ingenuity pathway analysis (IPA) version 9.0 (Ingenuity Systems, Redwood City, CA, USA) was used to analyze protein interaction networks and the functions of the serum proteins differentially expressed in hyperthyroidism patients. IPA software maps the UniProt IDs into the ingenuity knowledge base, the largest manually curated resource combining information from all published scientific studies. This software aids in determining the functions and pathways that are most strongly associated with the MS-generated protein list by overlaying the experimental expression data onto networks constructed from published interactions. A heat map that allows users to interactively visualize their data in the form of heat maps was created using Heatmapper, which is a freely available web server http://heatmapper.ca [50]. The identified proteins were additionally classified into different categories according to their molecular function and the biological processes in which they are involved; this was accomplished using the information in the GO database (http://www.geneontology.org/).

### 4.9. Immunoblotting

To validate the findings of the 2D-DIGE studies, three proteins with significantly different levels of abundance and connectivity in the network pathway were selected and examined through immunoblotting. Antibodies against haptoglobin (sc-69782 HP), apolipoprotein A1 (sc-376818 APOA1), and inter-alpha-trypsin inhibitor heavy chain 4 (sc-515353 ITIH4) were used. An equal amount of protein (5 µg) from each sample from the two groups (hyperthyroid, n = 10; euthyroid, n = 10) was taken, pooled together, and then separated by one-dimensional discontinuous slab gel electrophoresis on 12% SDS-polyacrylamide gels in duplicate [51]. Proteins were electrotransferred to Immobilon-P polyvinylidene difluoride (PVDF) transfer membranes (Millipore, Burlington, MA, USA) using a Mini Trans-Blot electrophoretic transfer cell (Bio-Rad, Hercules, CA, USA). Following transfer, the membranes were stained with Ponceau S to confirm transfer efficiency. The membranes were then blocked (5% fat free milk (FFM) in Tris-buffered saline (TBS), 1 h, room temperature) and rinsed three times with TBS-T (10 mM Tris HCl, 150 mM NaCl, 0.1% Tween 20). Membranes were then incubated with the appropriate primary antibody at its respective recommended dilution in blocking buffer, followed by incubation with the appropriate IgG- horseradish peroxidase (HRP)-conjugated secondary antibody. The immuno-reactive bands were detected by enhanced chemiluminescence (ECL, Thermo Fisher Scientific, Waltham, MA, USA), visualized by scanning with a FluorChem Q imager (Cell Biosciences, Palo Alto, CA, USA), and digitalized using the image analysis software AlphaView Q v3.0 (Cell Biosciences, Palo Alto, CA, USA).

## Figures and Tables

**Table 1:** Biochemical parameters of the hyperthyroid study subjects before and after carbimazole therapy. FT4, free thyroxine; TSH, thyroid-stimulating hormone; HDL, high-density lipoprotein; LDL, low-density lipoprotein.

	Hyperthyroid	Euthyroid	*p* value
**N**	10		
Age(years)	39.6 ± 10.6		
Glucose (mmol/L)	5.3 ± 0.8	5.2 ± 0.5	0.19
Urea (mmol/L)	3.9 ± 0.9	4.6 ± 0.8	0.18
Creatinine (umol/L)	61.1 ± 12.7	66.3 ± 12.0	0.39
Sodium (mmol/L)	138.6 ± 2.1	138.3 ± 0.9	0.16
Potassium (mmol/L)	4.2 ± 0.2	4.3 ± 0.4	0.40
Aspartate transaminase (IU/L)	34.8 ± 9.1	31.8 ± 4.5	0.38
Alanine transaminase (IU/L)	17.5 ± 5	15.8 ± 2.0	0.40
Alkaline phosphatase (IU/L)	114.6 ± 53.7	120.2 ± 28.9	0.30
FT4 (pmol/L)	35.4 ± 9.9	17.0 ± 2.8	0.001
TSH (mIU/L)	0.014 ± 0.01	0.8 ± 0.4	0.00068
Total cholesterol (mmol/L)	4.6 ± 1.0	4.9 ± 0.8	0.10
HDL cholesterol (mmol/L)	1 ± 0.2	1.4 ± 0.2	0.02
LDL cholesterol (mmol/L)	2.8 ± 0.9	3.1 ± 0.7	0.09
Triglycerides (mmol/L)	1.1 ± 0.6	0.9 ± 0.2	0.14

**Table 2:** List of the differentially expressed proteins identified in human plasma between hyperthyroid and euthyroid states, after treatment with antithyroid medication, using two-dimensional difference in gel electrophoresis (2D-DIGE). The differences in fold change are shown. Protein name, accession number, Mascot score, mass spectrometry (MS)% coverage, protein molecular weight (MW), and isoelectric point (pI) values according to the UniProt database are listed. ANOVA, analysis of variance.

Spot No	Accession No ^a^	Protein Name	MASCOT ID	Pi ^b^	MW ^c^	Cov%	Score ^d^	*p*-Value (ANOVA)	Ratio Hyper/Control	EXP ^e^
1153	P00738	**Haptoglobin**	HPT_HUMAN	6.13	45861	23	78	0.01	1.5	UP
729	P01876	**Ig alpha-1 chain C region**	IGHA1_HUMAN	6.08	38486	42	58	0.01	1.5	UP
1149	P00738	**Haptoglobin**	HPT _HUMAN	6.13	45861	27	69	0.01	1.7	UP
425	P13671	**Complement component C6**	CO6_HUMAN	6.39	108367	25	57	0.05	1.5	UP
966	O14791	**Apolipoprotein L1**	APOL1_HUMAN	5.60	44004	32	59	0.05	1.5	UP
855	P02750	**Leucine-rich alpha-2-glycoprotein**	A2GL_HUMAN	6.45	38382	42	71	0.05	1.5	UP
1097	P02647	**Apolipoprotein A-I**	APOA1_HUMAN	5.56	30759	72	238	0.05	1.5	DOWN
674	P02679	**Fibrinogen gamma chain**	FIBG_HUMAN	5.37	52106	50	102	0.012	1.5	UP
348	P00747	**Plasminogen**	PLMN_HUMAN	7.04	93247	30	77	0.016	1.8	DOWN
776	P01009	**Alpha-1-antitrypsin**	A1AT_HUMAN	5.37	46878	40	78	0.039	1.7	DOWN
965	P01009	**Alpha-1-antitrypsin**	A1AT_HUMAN	5.37	46878	36	68	0.047	1.5	DOWN
958	P10909	**Clusterin**	CLUS_HUMAN	5.89	53031	26	78	0.053	1.5	UP
763	P02675	**Fibrinogen beta chain**	FIBB_HUMAN	8.54	56577	55	205	0.050	1.5	DOWN
435	P00736	**Complement C1r subcomponent**	C1R_HUMAN	5.82	81606	26	99	0.051	1.7	DOWN
780	P00736	**Complement C1r subcomponent**	C1R_HUMAN	5.82	81606	33	115	0.0268	1.8	DOWN
560	Q14624	**Inter-alpha-trypsin inhibitor heavy chain H4**	ITIH4_HUMAN	6.51	103521	37	85	0.0386	1.5	DOWN
1339	P04217	**Alpha-1B-glycoprotein**	A1BG_HUMAN	5.58	54809	37	90	0.016	1.5	UP
1591	P02679	**Fibrinogen gamma chain**	FIBG_HUMAN	5.37	52106	50	129	0.0372	1.5	UP
849	P15169	**Carboxypeptidase N catalytic chain**	CBPN_HUMAN	6.86	52538	33	62	0.021	1.5	UP
835	P02790	**Hemopexin**	HEMO_HUMAN	6.55	52385	48	123	0.025	1.5	UP

^a^ Protein accession number for SWISSPROT Database. ^b^ Theoretical isoelectric point. ^c^ Theoretical relative mass. ^d^ MASCOT score. ^e^ Protein expression between hypothyroid and euthyroid states.

## Supplementary Materials

The following are available online, Figure S1. Pathways and canonical pathways identified in the IPA functional analysis. Table S2. Dye-switching strategy applied during labeling to avoid dye-specific bias. A total of 20 patient samples were run on 10 2D-PAGE gels. Samples were labeled randomly with Cy3 and Cy5, and a pooled sample was used as an internal standard and stained with Cy2. Table S1. Table of statistical data of the mean expression level of the significant proteins before and after treatment given as mean log normalized volume. Figure S2. DIGE gel images for all the samples labeling according to Table S2.
