# Supplementary material for: Plasma-Based Proteomics Profiling of Patients with Hyperthyroidism after Antithyroid Treatment
Source: Molecules. 2020 Jun 19;25(12):2831. doi: 10.3390/molecules25122831 (PMC7356574; doi:10.3390/molecules25122831)

GEL-1 CY3: EUTHYROID (S:1)

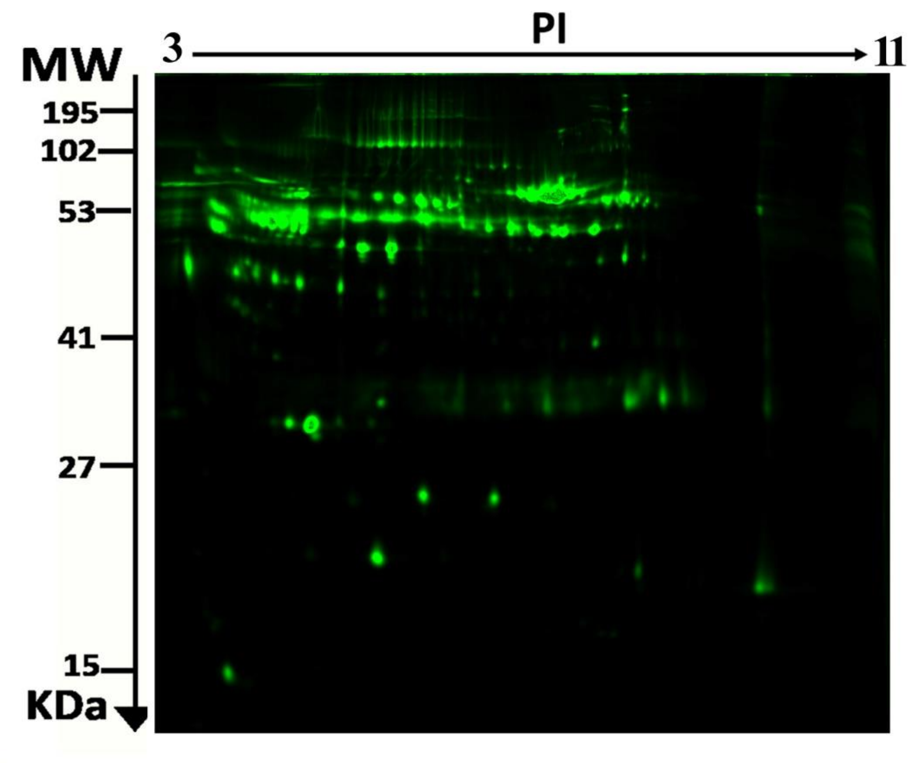

GEL-1 CY5: HYPERTHYROID (S:11)

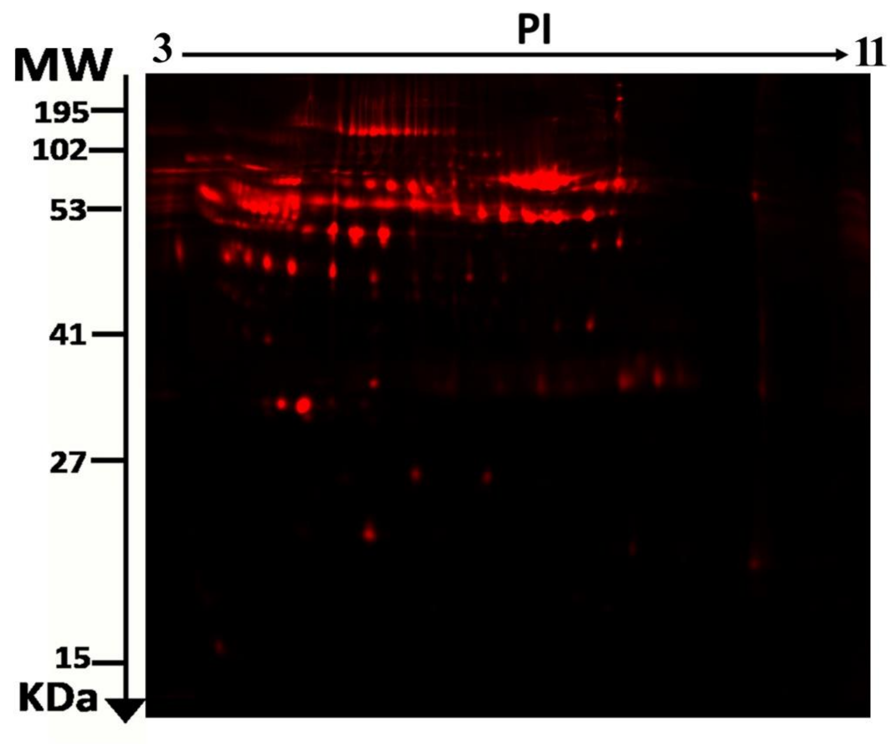

GEL-2 CY3: HYPERTHYROID (S:12)

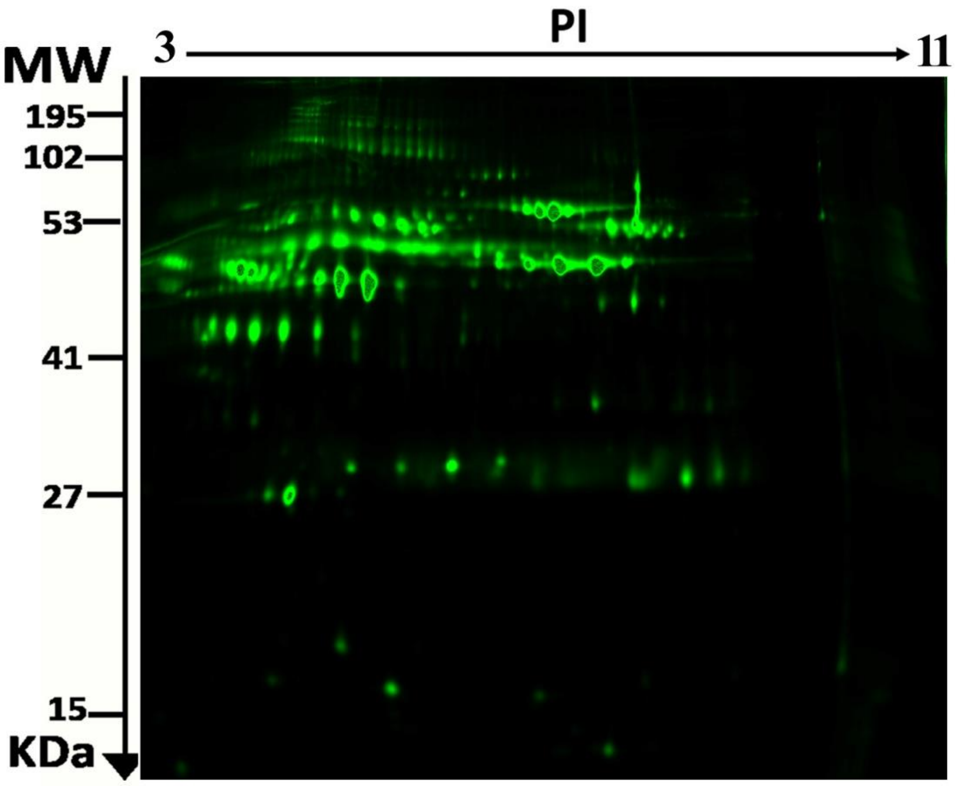

GEL-2 CY5: EUTHYROID (S:2)

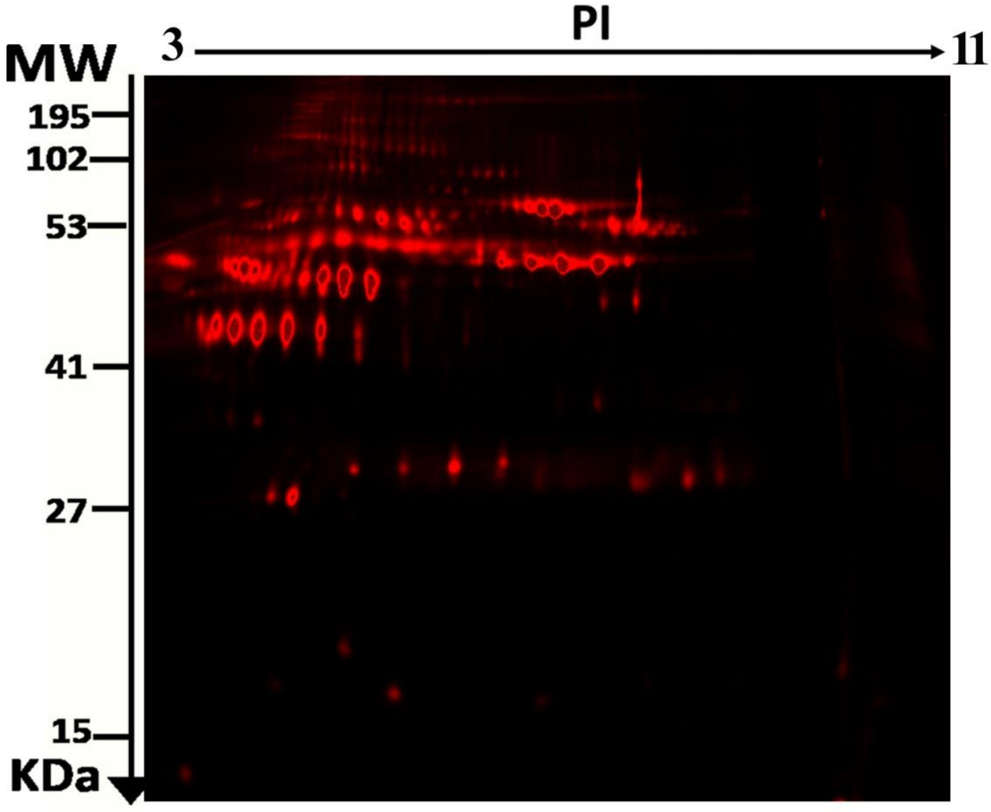

GEL-3 CY3: EUTHYROID (S:5)

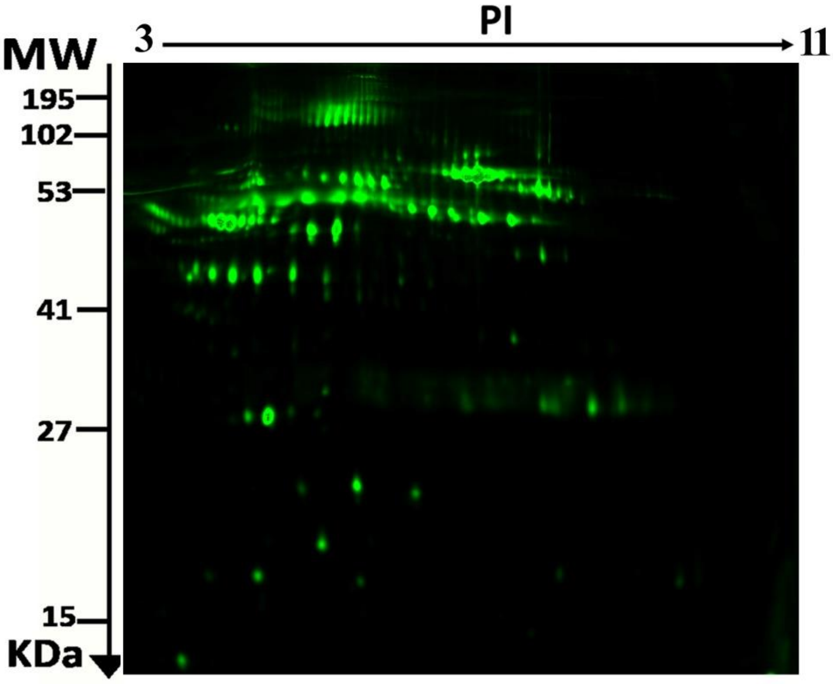

GEL-3 CY5: HYPERTHYROID (S:13)

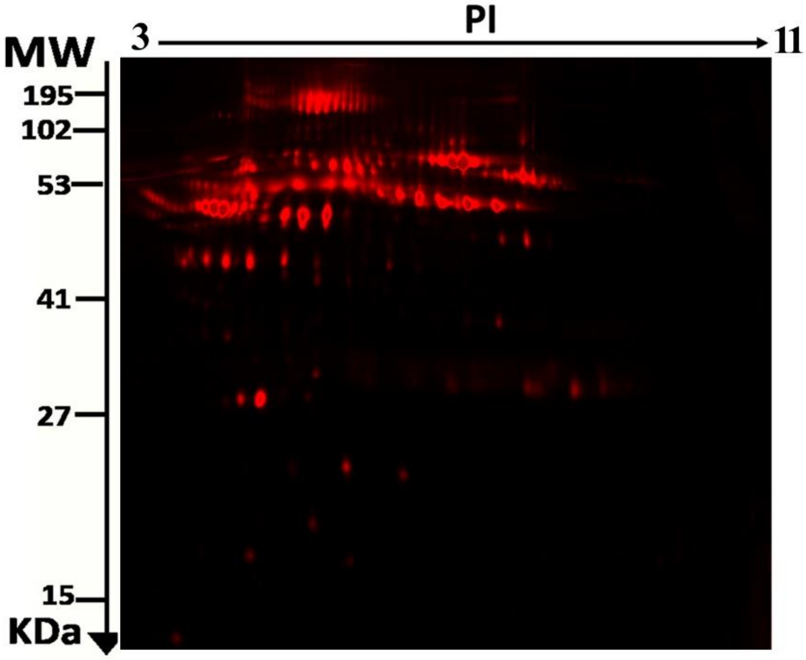

GEL-4 CY3: HYPERTHYROID (S:14)

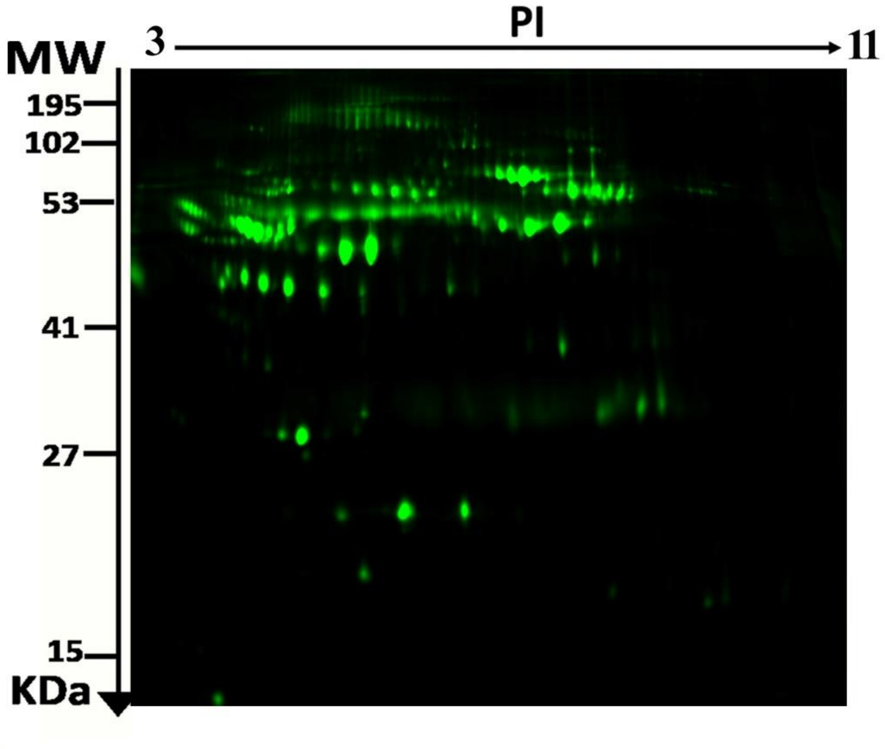

GEL-4 CY5: EUTHYROID (S:3)

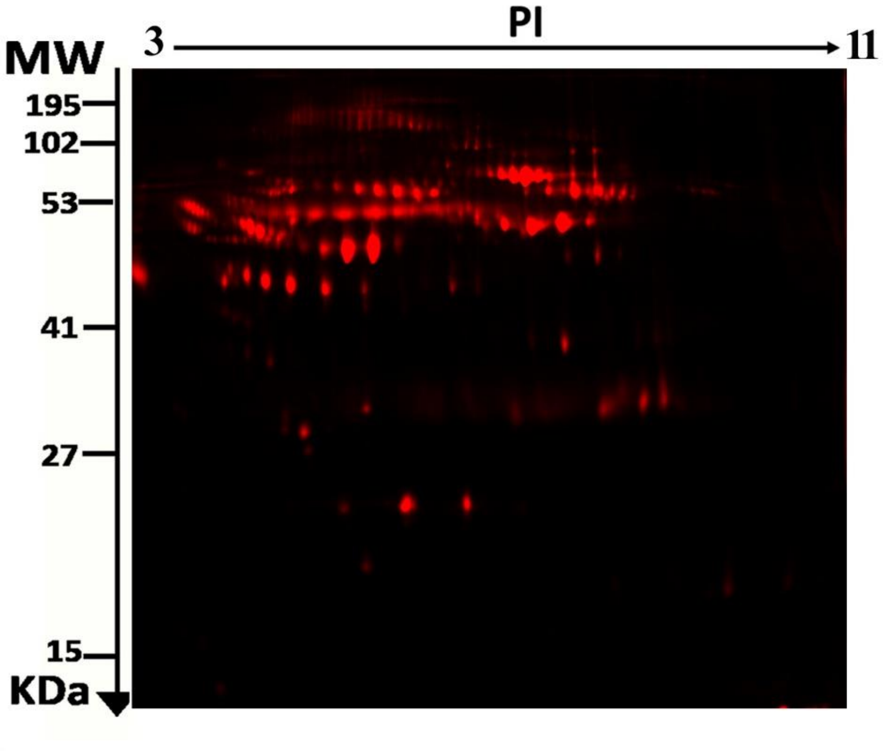

GEL-5 CY3: HYPERTHYROID (S:15)

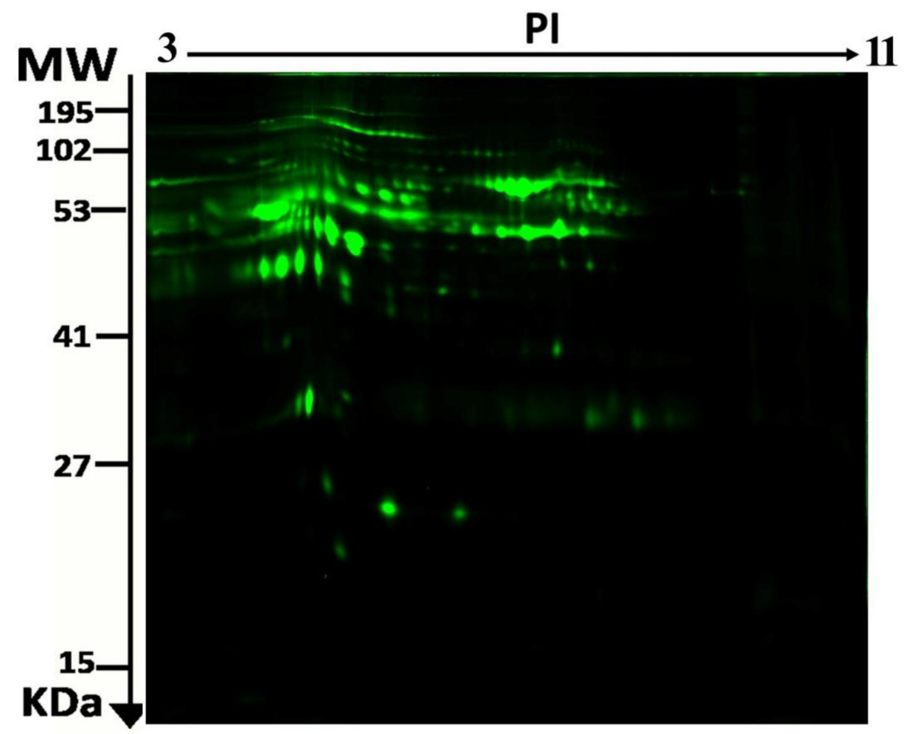

GEL-5 CY5: EUTHYROID (S:6)

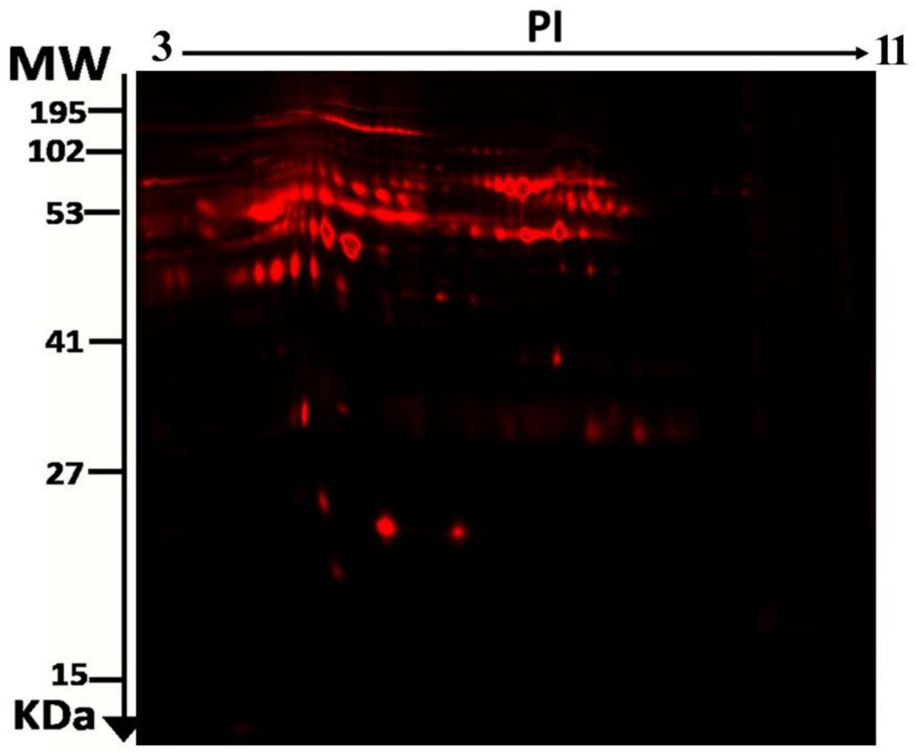

GEL-6 CY3: HYPERTHYROID (S:16)

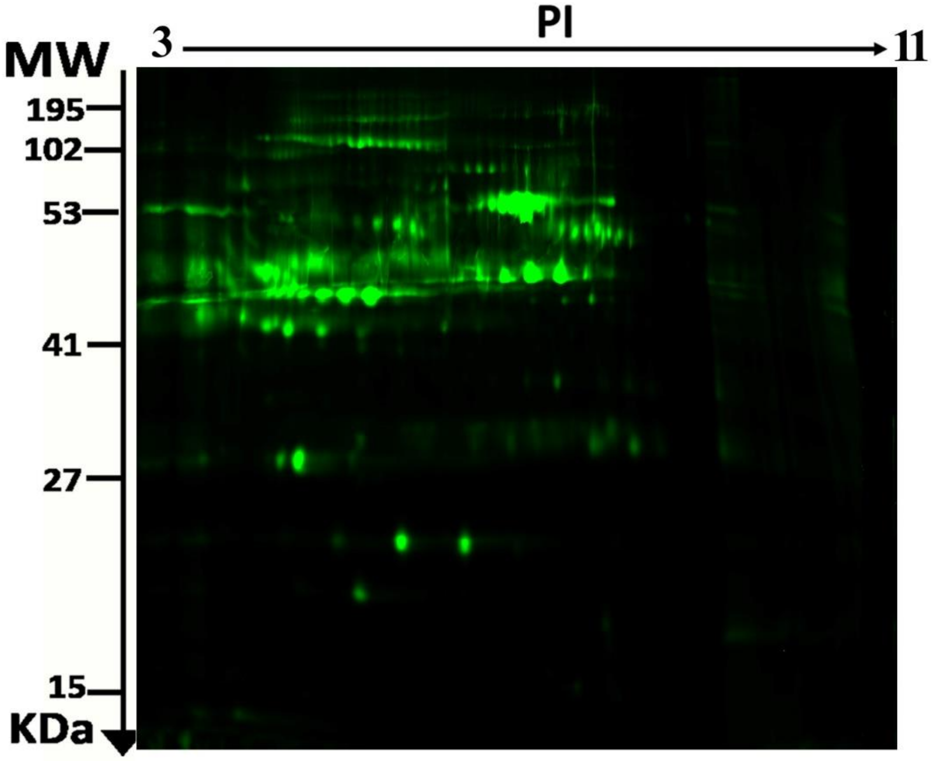

GEL-6 CY5: EUTHYROID (S:4)

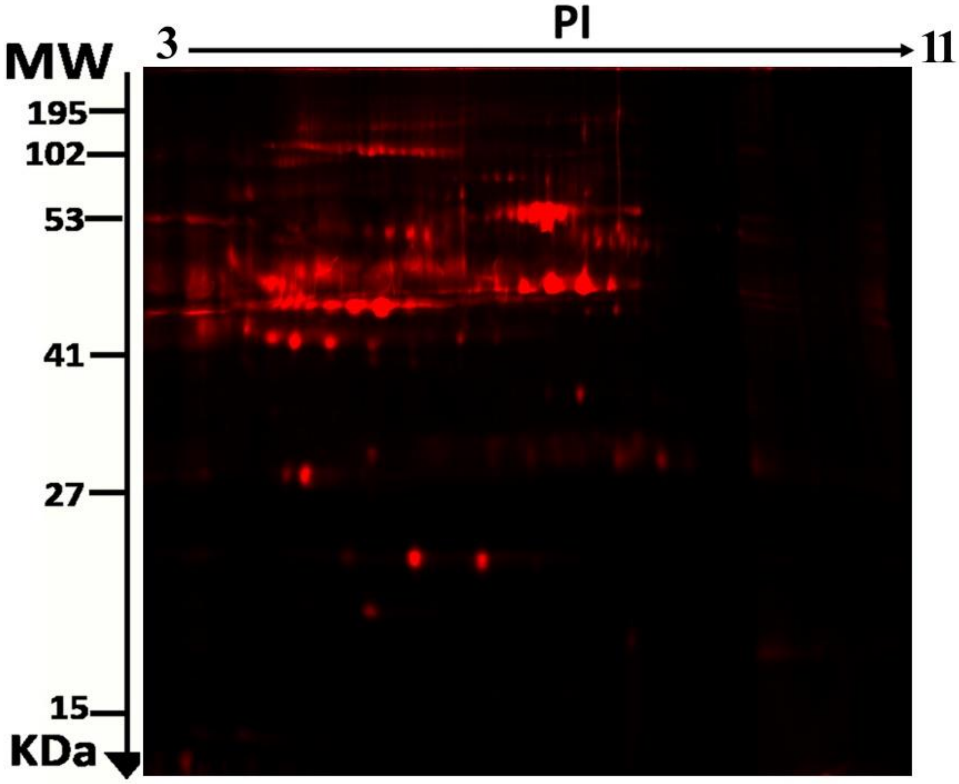

GEL-7 CY3: EUTHYROID (S:7)

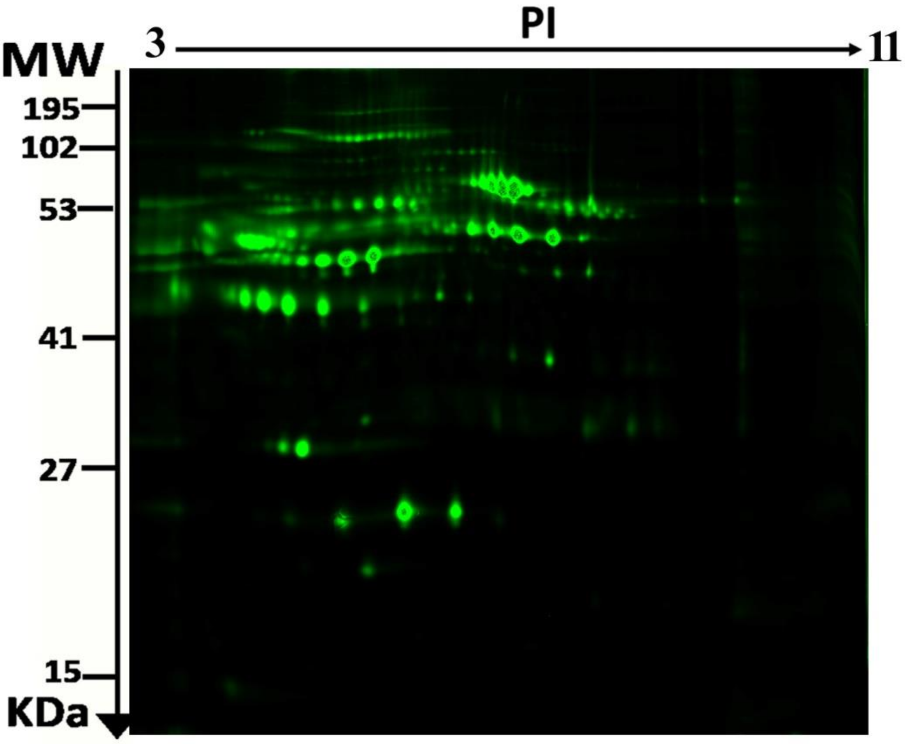

GEL-7 CY5: HYPERTHYROID (S:17)

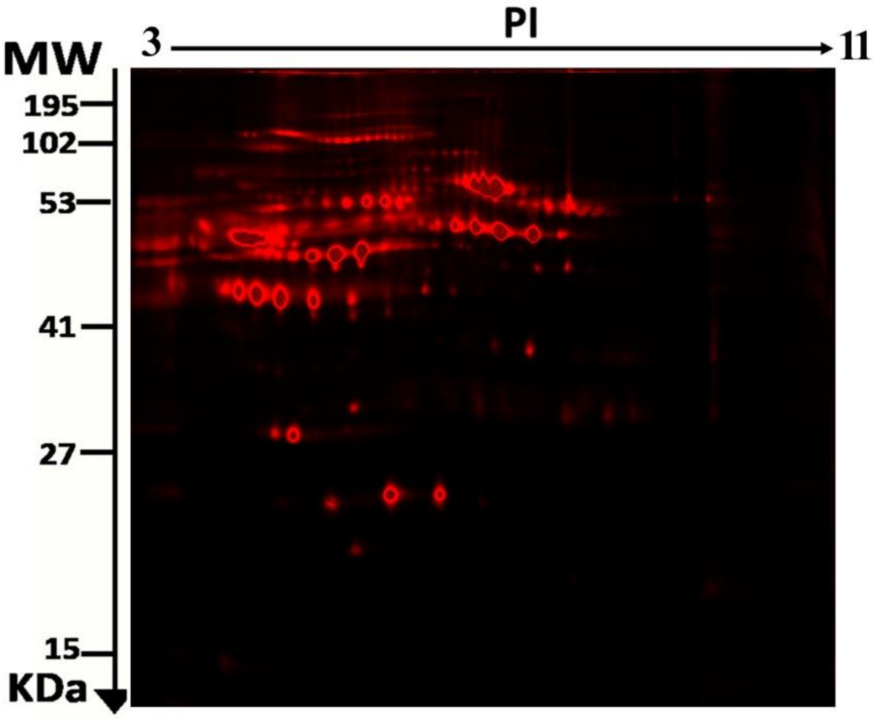

GEL-8 CY3: HYPERTHYROID (S:18)

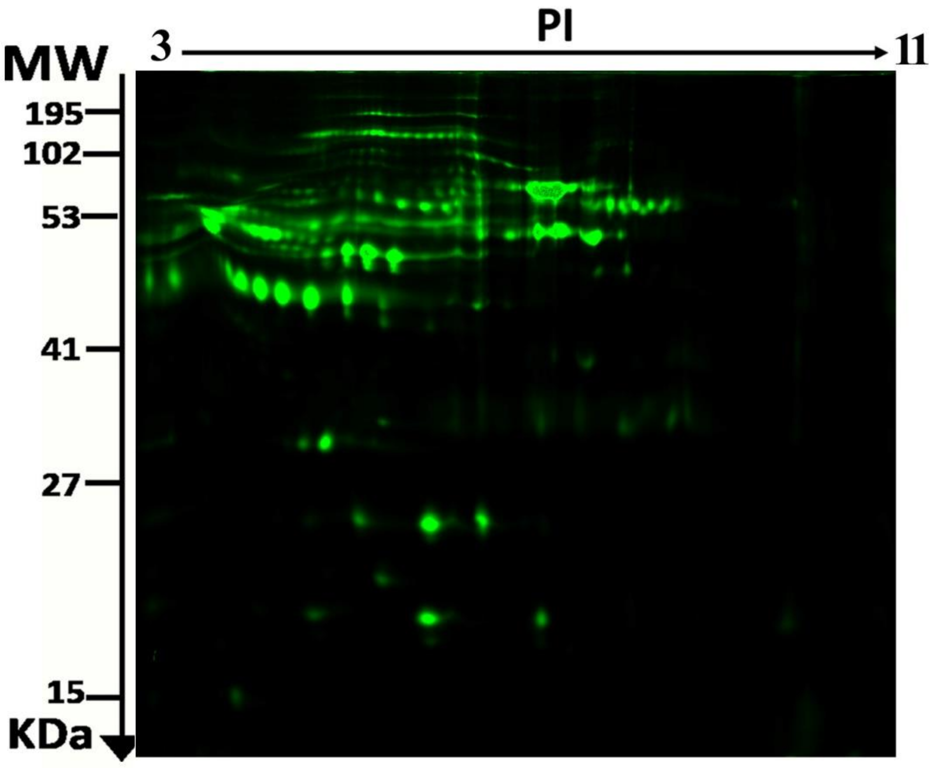

GEL-8 CY5: EUTHYROID (S:8)

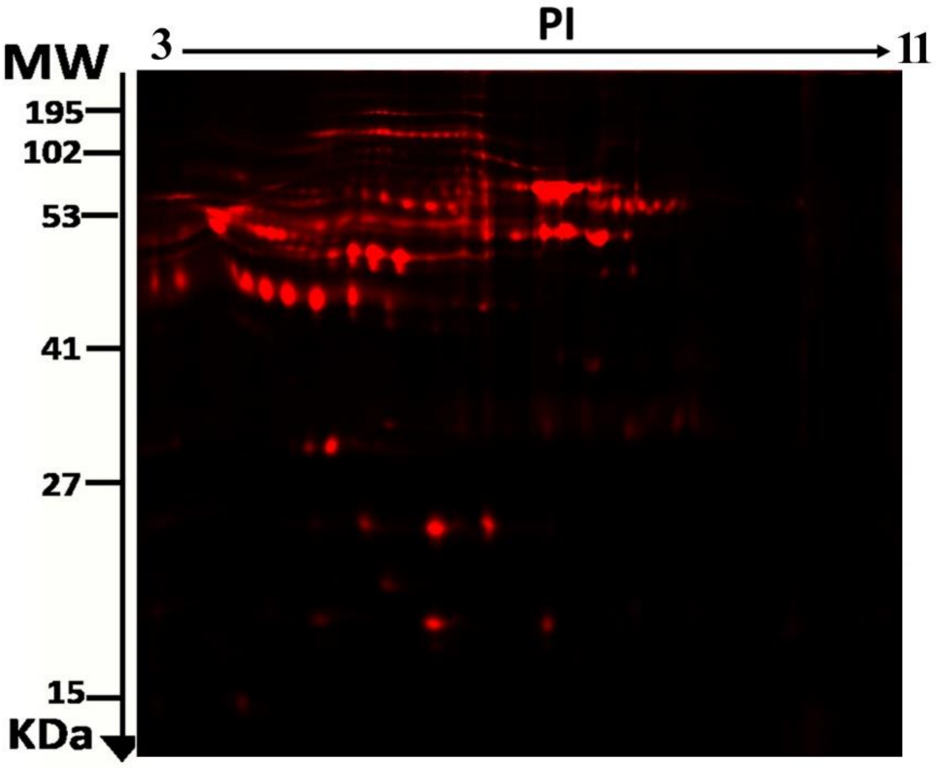

GEL-9 CY3: EUTHYROID (S:9)

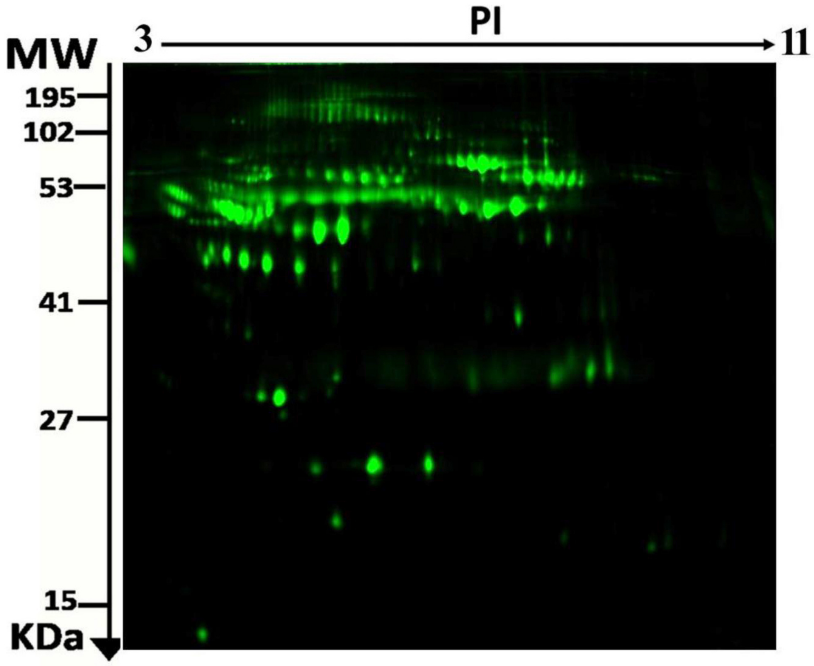

GEL-9 CY5: HYPERTHYROID (S:19)

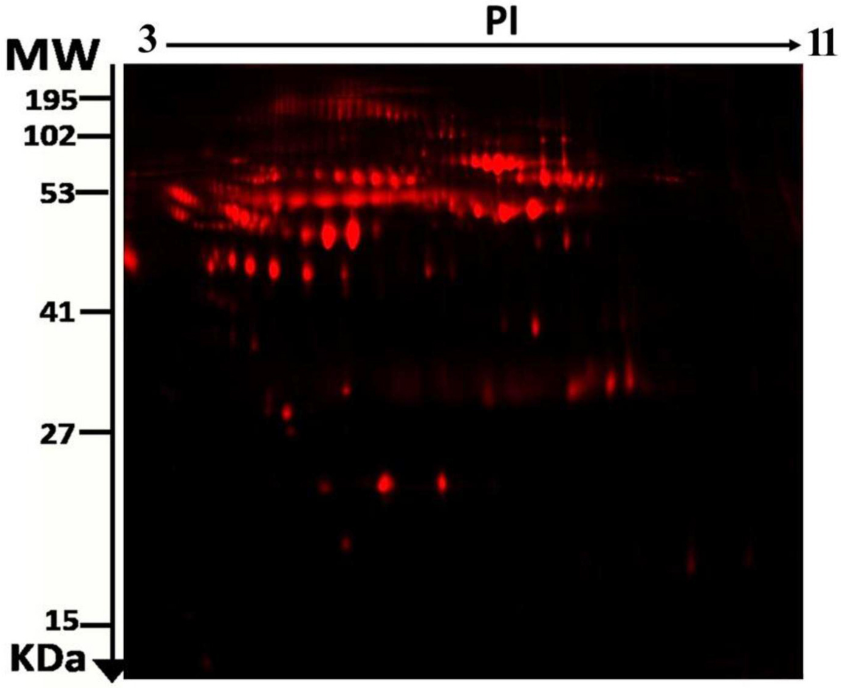

GEL-10 CY3: HYPERTHYROID (S:20)

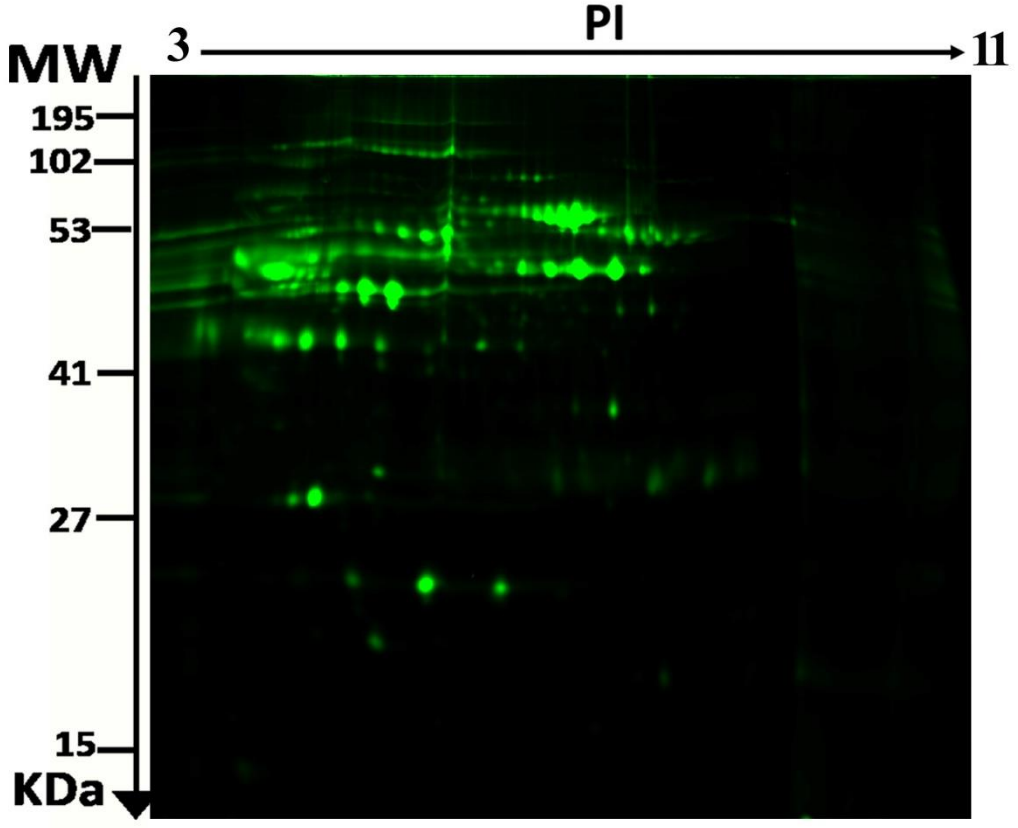

GEL-10 CY5: EUTHYROID (S:10)

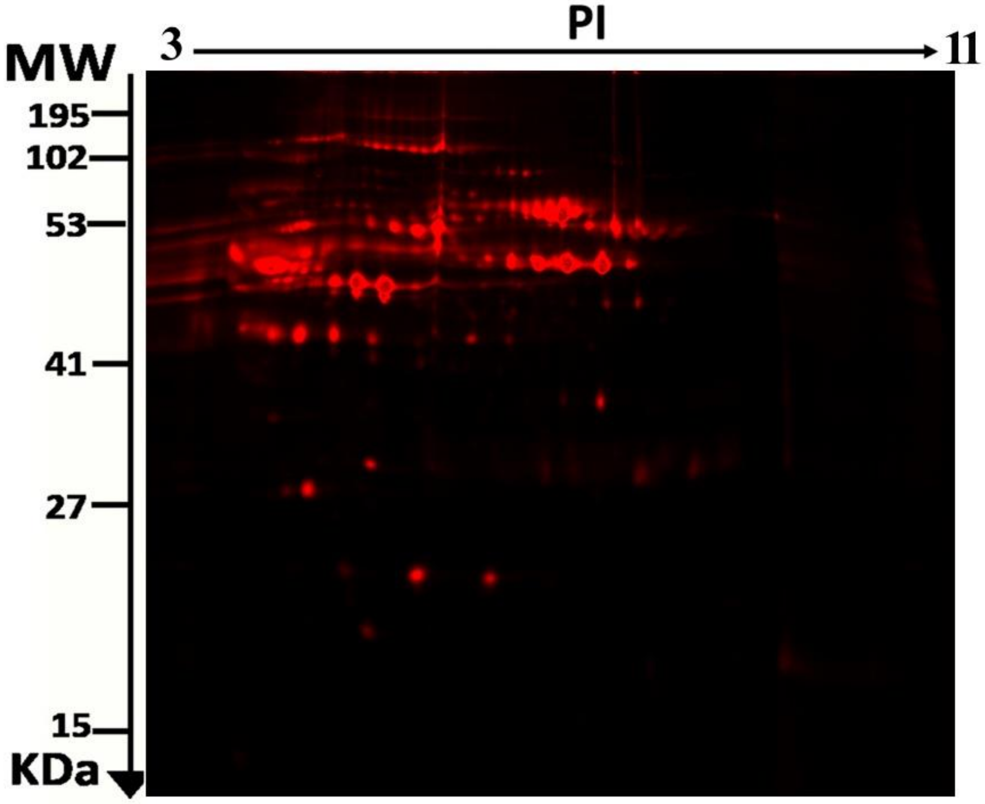

Supplement: Supplementary file 1 [file molecules-25-02831-s001.zip › Figure S2.pdf]
