# Supplementary material for: Plasma-Based Proteomics Profiling of Patients with Hyperthyroidism after Antithyroid Treatment
Source: Molecules. 2020 Jun 19;25(12):2831. doi: 10.3390/molecules25122831 (PMC7356574; doi:10.3390/molecules25122831)
Supplement: Supplementary file 1 [file molecules-25-02831-s001.zip › Supplementary File.pdf]

Supplementary File

Figure S1: Pathways and canonical pathways identified in the IPA functional analysis.

Top Canonical Pathways

Name	p-value	Overlap	
Acute Phase Response Signaling	9.94E-14	4.7 %	8/169
LXR/RXR Activation	1.65E-10	5.0 %	6/121
FXR/RXR Activation	2.11E-10	4.8 %	6/126
Coagulation System	9.08E-09	11.4 %	4/35
Atherosclerosis Signaling	1.62E-06	3.2 %	4/125

Top Networks

ID	Associated Network Functions	Score
1	Infectious Diseases, Inflammatory Disease, Organismal Injury and Abnormalities	31
2	Cell-To-Cell Signaling and Interaction, Cellular Compromise, Cellular Function and Maintenance	3

Table S2: Dye-switching strategy applied during labeling to avoid dye-specific bias. A total of 20 patient samples were run on 10 2D-PAGE gels. Samples were labeled randomly with Cy3 and Cy5, and a pooled sample was used as an internal standard and was stained with Cy2.

Gel	Cy3	Cy5	Cy2
1	1 (Euthyroid)	11 (Hyperthyroid)	Pooled sample
2	12 (Hyperthyroid)	2 (Euthyroid)	Pooled sample
3	5 (Euthyroid)	13 (Hyperthyroid)	Pooled sample
4	14 (Hyperthyroid)	3 (Euthyroid)	Pooled sample
5	15 (Hyperthyroid)	6 (Euthyroid)	Pooled sample
6	16 (Hyperthyroid)	4 (Euthyroid)	Pooled sample
7	7 (Euthyroid)	17 (Hyperthyroid)	Pooled sample
8	18 (Hyperthyroid)	8 (Euthyroid)	Pooled sample
9	9 (Euthyroid)	19 (Hyperthyroid)	Pooled sample
10	20 (Hyperthyroid)	10 (Euthyroid)	Pooled sample
