# Supplementary material for: Plasma-Based Proteomics Profiling of Patients with Hyperthyroidism after Antithyroid Treatment
Source: Molecules. 2020 Jun 19;25(12):2831. doi: 10.3390/molecules25122831 (PMC7356574; doi:10.3390/molecules25122831)
Supplement: Supplementary file 1 [file molecules-25-02831-s001.zip › Table S1.pdf]

Spots

Spot No	Anova (p)	Fold	Average Normalised Volumes	
			EUTH	HYPER
849	0.021	1.5	8.031 e+005	1.202 e+006
835	0.025	1.5	7.414 e+005	1.112 e+006
776	0.039	1.7	1.307 e+006	7.682 e+005
965	0.047	1.5	1.024 e+006	6.828 e+005
763	0.050	1.5	1.248 e+006	8.310 e+005
674	0.012	1.5	7.574 e+005	1.136 e+006
348	0.016	1.8	1.760 e+006	9.561 e+005
435	0.051	1.7	1.370 e+006	8.080 e+005
1097	0.050	1.5	7.673 e+006	5.115 e+006
966	0.050	1.5	1.005 e+007	1.509 e+007
855	0.050	1.5	8.126 e+006	1.219 e+007
425	0.050	1.5	4.062 e+005	6.092 e+005
1149	0.011	1.7	1.361 e+007	2.302 e+007
729	0.012	1.5	1.254 e+007	1.888 e+007
1153	0.012	1.5	1.582 e+007	2.383 e+007
560	0.039	1.5	1.730 e+006	1.153 e+006
1339	0.016	1.5	7.683 e+005	1.152 e+006
1591	0.037	1.5	1.034 e+006	1.543 e+006
958	0.053	1.5	7.202 e+005	1.081 e+006
780	0.027	1.8	5.983 e+005	3.400 e+005
